# Copper Oxide Nanoparticles Cause a Dose-Dependent Toxicity via Inducing Reactive Oxygen Species in Drosophila

**DOI:** 10.3390/nano8100824

**Published:** 2018-10-12

**Authors:** Eugene Baeg, Kanidta Sooklert, Amornpun Sereemaspun

**Affiliations:** 1 Daegu International School, 22 Palgongro, 50-Gil, Donggu, Daegu 701-170, Korea; eugenebaeg@gmail.com; 2 Nanomedicine Research Unit, Department of Anatomy, Faculty of Medicine, Chulalongkorn University, Bangkok 10330, Thailand; kanidta.s1986@gmail.com

**Keywords:** copper oxide nanoparticle, cytotoxicity, reactive oxygen species, Nrf2, Drosophila melanogaster

## Abstract

Copper oxide nanoparticles (CuONPs) have attracted considerable attention, because of their biocide potential and capability for optical imaging, however CuONPs were shown to be highly toxic in various experimental model systems. In this study, mechanism underlying CuONP-induced toxicity was investigated using *Drosophila* as an in vivo model. Upon oral route of administration, CuONPs accumulated in the body, and caused a dose-dependent decrease in egg-to-adult survivorship and a delay in development. In particular, transmission electron microscopy analysis revealed CuONPs were detected inside the intestinal epithelial cells and lumen. A drastic increase in apoptosis and reactive oxygen species was also observed in the gut exposed to CuONPs. Importantly, we found that inhibition of the transcription factor Nrf2 further enhances the toxicity caused by CuONPs. These observations suggest that CuONPs disrupt the gut homeostasis and that oxidative stress serves as one of the primary causes of CuONP-induced toxicity in *Drosophila*.

## 1. Introduction

Nanoparticles (NPs) have unique physicochemical properties caused by their small size, ranging from 1 and 100 nm, and high surface to volume ratio. Hence, applications of manufactured NPs in consumables and biomedical devices are continuously expanding while the increasing use of NPs is associated with potential adverse health concerns [1]. Copper-based NPs have attracted an increased attention for biomedical and industrial applications. For example, smaller CuONPs are fluorescent and have capability for optic imaging, which enables CuONPs to serve as a versatile vehicle for drug delivery and image-guided therapy [2]. CuONPs also show significant antibacterial and biocidal activities, and thus they are being used for the development of many dental and surgical instruments, such as dental composite [3,4,5]. Furthermore, CuONPs have unique physicochemical properties, such as high electrical and temperature conductivity. Hence, they have been used in magnetic storage media, thermal and electrical devices, sensors, catalysis and semiconductors [6,7,8,9]. However, this wide-scale use of CuONPs makes us more prone to their exposure and their potential adverse health effects. In addition, their frequent use provides a negative impact on aquatic ecosystems.

Inhalation is one of the most common routes of metal oxide NP exposure. In the murine model of ovalbumin-induced asthma, CuONPs showed to increase airway hyper-responsiveness, inflammation-related cell number and pro-inflammatory cytokine expression [10], suggesting the pulmonary toxicity of CuONPs. In support of this, recent studies showed that CuONPs facilitate pulmonary epithelial cell death and inflammation, eventually leading fibrosis in the C57BL/6 mice model [11]. Furthermore, CuONP exposure was shown to induce fibrotic responses in the mouse respiratory track via up-regulating the expression of transforming growth factor-β1 (TGF-β1) and phosphorylation of Smad3, accompanying with an induction of proinflammatory cytokines, such as interleukin (IL)-1β, IL-6 and tumor necrosis factor-α (TNF-α) [12]. CuONPs have also shown to cause toxic effects in other model organisms. For instance, zebrafish exposed to CuONPs exhibited defects in the liver, neural and cardiac development, dorsoventral patterning and retinal neuro-differentiation [13,14]. Acute genotoxic effects and degeneration of dopaminergic neurons by CuONPs were also reported in the fruit fly *Drosophila* and *C. elegans* model, respectively [15,16,17]. Cytotoxic effects of CuONPs were also reported in various in vitro cellular models. In the human keratinocyte ski HaCaT cells exposed to CuONPs, caspase-3 activity was drastically up-regulated, suggesting that CuONPs can cause apoptosis [18]. CuONPs are also known to modulate various cellular responses such cell cycle arrest, autophagy and apoptosis in the breast cancer MCF7 cells [19]. Furthermore, CuONPs showed a dose-dependent cyto- and geno-toxicity in the human lung epithelial A549 cells by inducing DNA damage [20], together with a significant reduction in the antioxidant glutathione and induction in caspase-3 activity. Treatment of cultured primary brain astrocytes with CuONPs (~5 nm in diameter) was shown to compromise the cell viability in time- and dose-dependent manners [21].

Metal oxide nanoparticles are well known to generate oxidative stress and deregulate normal cellular activities, which subsequently leads to cellular toxicity. Hence, oxidative stress has been considered as one of the primary causes of nanotoxicity and has been reported to use as bio-indicator to evaluate the toxic effects of nanoparticles [22]. In support of this, CuONP exposure was shown to cause a significant accumulation of intracellular reactive oxygen species (ROS) in various cultured cell models and marine model species, such as zebrafish and shrimp [23]. Importantly, quantitative metabolomics and biochemical approaches have revealed that CuONPs induce the regulation of metabolites, such as cysteine-glutathione disulfide and citrulline, which are related to oxidative stress in A549 cells [24], and that the expression and activity of various antioxidant/detoxifying enzymes, such as glutathione peroxidase, superoxide dismutase, glutathione S-transferase, lipid peroxidase and catalase are altered in the mouse hippocampal HT22 cells [25,26]. Furthermore, the addition of the antioxidant NAC (*N*-acetyl-cysteine) to kidney epithelial cells was shown to mitigate CuONP-induced DNA damage and cell death [27], suggesting the critical role of oxidative stress in CuONP-mediated toxicity. However, direct evidence demonstrating that aberrant ROS are induced by CuONPs in living organism and modulating the ROS levels can indeed modulate toxicity in vivo is still lacking.

The fruit fly *Drosophila* has a short life cycle of approximately 10–12 days at ambient temperature, distinct development stages; embryonic, larval, pupal and adult stages, high levels of physiological and structural similarity to humans, and a less genetic redundancy compared to mammals. Furthermore, numerous genetic tools and reagents, such as tens of thousands mutant and transgenic lines are easily available for characterizing and dissecting outstanding biological questions. In support of this, *Drosophila* has received extensive attention for the past decade as a reliable in vivo model organism to understand the fundamental biology of NP-induced toxicity. In this study, we used the fruit fly *Drosophila* as an in vivo model to examine the potential toxic effects of CuONPs and investigate the underlying mechanism. We found that upon oral route of exposure CuONPs result in a dose-dependent decrease in the egg-to-adult survivorship and delay in development. In particular, ingested CuONPs were detected in the intestinal epithelial cells, which is associated with a drastic induction of cell death and ROS in the gut. Importantly, genetic manipulation of the transcriptional factor Nrf2 (Nuclear factor erythroid 2–related factor 2) activity suggested that intracellular ROS is one of the essential factors that determines the *Drosophila* viability.

## 2. Materials and Methods

### 2.1. Characterization of CuONPs

CuONPs (Sigma-Aldrich; CAS no. 1317-38-0, Darmstadt, Germany) of particle size less than 50 nm (by dynamic light scattering [DLS]) were used in this study. The nanoparticle stock solutions were then diluted to 1 mg/mL and sterile-filtered before using for assays. The morphology of the CuONPs were studied using transmission electron microscopy (TEM) (JEOL JEM 1010, Tokyo, Japan). TEM samples were prepared by dropping colloidal CuONPs on copper grids pre-coated with Formvar. The Zetasizer Nano was also used to measure the surface charge of the CuONPs.

### 2.2. Fly Strains

Fly stocks were maintained under standard culture conditions. w1118 (a white eyed stock) fly strain was obtained from the Bloomington *Drosophila* Stock Center and used for toxicity assays, including egg-to-adult survivorship and development. GstD1-green fluorescent protein (GFP) fly line obtained from D. Bohmann was used to monitor the effect of CuONPs on intracellular ROS induction in the gut. To determine whether inhibition of CncC (the *Drosophila* homolog of Nrf2), a key transcriptional factor responsible for the expression of antioxidants and detoxifying enzymes, can further increase CuONP-induced intracellular ROS levels and decrease the CuONP-induced toxicity, virgin female CncCVL110/TM3, Sb flies (a gift from D. Bohmann) were crossed with male w1118 flies. Flies were transferred to food containing 0, 0.05 or 0.1 mg/mL CuONPs. Eclosed F1 adults were collected and counted, and the percentage of viability with respect to control flies with balancer (TM3, Sb allele) was calculated.

### 2.3. Drosophila Exposed to CuONPs

CuONPs at the concentrations of 0, 0.05, 0.1, 0.15 or 0.25 mg/mL were added to *Drosophila* food, which consists of cornmeal flour, dextrose, brewer’s yeast, Bacta agar and Nipagin. *Drosophila* food without CuONPs (0 mg/mL) was used as a control. Both parental male and female flies were added to vials with or without CuONPs. The flies were then allowed to mate for five days, and then removed. Newly laid eggs (F1 progenies) were maintained in the presence of CuONPs at different concentrations until they emerged (embryonic stage to adult stage).

### 2.4. Viability and Development of Drosophila upon CuONPs Treatment

The effects of CuONPs on viability were evaluated by counting the number of successfully eclosed F1 flies. The number of days required for the first eclosion was recorded to study the effects of CuONPs on developmental process. Three independent experiments were carried out for statistical analysis.

### 2.5. Inductively Coupled Plasma-Mass Spectrometry

To quantify the amount of CuONPs accumulation in *Drosophila*, inductively coupled plasma-mass spectrometry (ICP-MS) (Perkin Elmer, USA) was performed. CuONP-fed third instar larvae were homogenized in ultrapure water using a homogenizer to eliminate any source of salt or minerals, which may confound the subsequent analysis and then acid-digested for 24 h by Aqua Regia. The samples were then diluted with deionized water to appropriate volumes using the Agilent 7500 Series ICP-MS (Perkin Elmer, Norwalk, CT, USA). The final nitric acid was not more than 2%. Three independent experiments were carried out.

### 2.6. TEM Study

To show the accumulation of NPs in the third instar larval intestine upon CuONP ingestion, untreated and CuONP-treated third instar larval midguts were dissected out and collected in phosphate-buffered saline (PBS) for TEM preparation. In brief, the midguts were fixed in 2.5% glutaraldehyde for 1 h, and subsequently osmified with 1% OsO4 and bits of KFeCN at room temperature for 1 h. Samples were then dehydrated and embedded in epoxy resin (polymerization at 60 °C for overnight) (Ted Pella Inc., Redding, CA, USA), followed by slicing of ultrathin sections. The sections were subsequently stained with lead citrate (BDH, Bristol, UK). Digital micrographs were obtained using a Gatan 792 Bioscan 1 k × 1 k Wide Angle Multiscan charge-coupled device camera attached to the JEOL 1010 TEM (Tokyo, Japan).

### 2.7. Cellular ROS Detection Assay

In *Drosophila*, CncC regulates the expression of gstD1 (glutathione S transferase D1), which encodes a detoxification enzyme. To monitor the effects of CuONPs on ROS induction in the gut, transgenic flies carrying an oxidative stress reporter gene gstD1-GFP were used to assess whether CuONP exposure can induce the oxidative stress reporter activity. The third instar larval midguts were dissected in dissecting solution pH 7.2 (130 mM NaCl; 1.9 mM CaCl_2_; 4.7 mM KCl; 10 mM HEPES) and observed under the confocal microscope (Olympus Fluoview FV1000 cLSM, Tokyo, Japan) to monitor GFP expression. To further confirm the induction of ROS upon CuONPs, the dissected guts were also stained with 10 μM dihydroethidium (DHE) after fixation for 5 min and then washed in PBST three times. The guts were then mounted on Vectashield mounting medium containing DAPI on a slide glass and viewed under the confocal microscope.

### 2.8. Terminal Deoxynucleotidyl Transferase dUTP Nick End Labelling (TUNEL)-Assay of the Drosophila Gut

The guts treated with or without CuONPs were subjected to Terminal deoxynucleotidyl transferase dUTP nick end labelling (TUNEL) assay (catalog number 11684795910; Roche Applied Science, Mannheim, Germany). Briefly, samples were fixed in 4% paraformaldehyde, permeabilized with 0.1% Triton X-100 in 0.1% sodium citrate solution followed by labeling with TUNEL reaction mixture.

### 2.9. Statistical Analysis

Statistical analyses were performed using the GraphPad Prism software (version 6.0). Values from all experiments were expressed in mean ± standard error. The data was analyzed by unpaired *t*-test or one-way ANOVA with post-hoc test (Tukey’s Multiple Comparison Test). *p* < 0.05 was statistically significant.

## 3. Results and Discussion

### 3.1. Characterization of CuONPs

**Figure 1:**
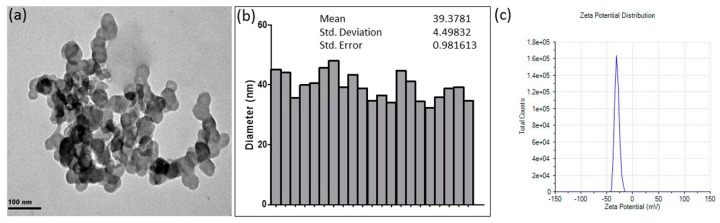
Characterization of Copper oxide nanoparticles (CuONPs). (**a**) Transmission electron microscopy (TEM) micrographs show spherical CuONPs with the size of approximately 40 nm; (**b**) The size distribution of CuONPs was determined by ImageJ software; (**c**) The zeta potential of CuONPs is −28.1 mV.

The size and morphology of CuONPs used in this study were examined using transmission electron microscopy (TEM). TEM micrographs showed that CuONPs are spherical in shape and uniform homogenously dispersed in suspension, with the defined size of approximately 40 nm (Figure 1a). Consistently, the size distribution of CuO particles, which was determined by ImageJ software showed that the average diameter of CuONPs is ~40 nm, as observed under TEM (Figure 1b). Lastly, the zeta potential of the CuONPs was found to be −28.1 mV, suggesting that they are relatively stable (Figure 1c). As shown in Figure 1a, agglomeration of CuONPs was observed, and thus freshly sonicated CuONP dispersions were used immediately for assays.

### 3.2. CuONPs Cause Toxic Effects in the Fruit Fly Drosophila

**Figure 2:**
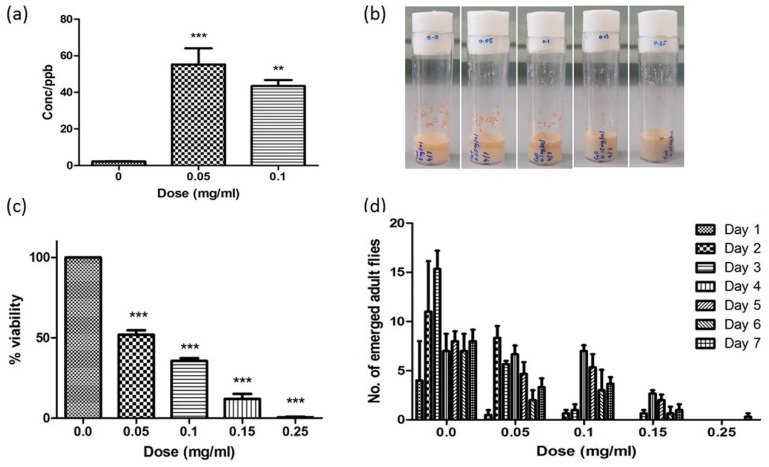
CuONP ingestion induces toxic effects in *Drosophila*. (**a**) CuONP-fed F1 progenies show an uptake and accumulation of CuONPs in the body. (**b**,**c**) A dose-dependent decline in the number of pupa and in egg-to-adult survivorship is observed upon CuONPs. (**d**) CuONP exposure causes a dose-dependent delay in development. Error bar = SEM, * *p*-value < 0.05; ** *p*-value < 0.01; *** *p*-value < 0.001.

To examine the potential toxic effects of CuONPs with the size of ~40 nm in *Drosophila*, we first monitored the uptake and accumulation of CuONPs in vivo after oral route of administration. F1 (first filial or generation) progenies derived from the control parental w1118 flies were maintained in food in the presence of CuONPs at various concentrations, ranging from 0.05 to 0.1 mg/mL. We then performed the inductively coupled plasma mass spectrometry (ICP-MS) analysis using CuONP-fed third instar larvae to show that CuONPs indeed accumulate in the body. Five late third instar larvae were collected from each experimental group, homogenized, and then acid-digested. The concentration of CuONPs per parts per billion (ppb), which indicates the number of units of mass of a contaminant per 1000 million units of total mass was determined. As shown in Figure 2a, upon exposure to CuONPs at the concentration of 0.05 mg/mL, a drastic accumulation of CuONPs was observed compared to control. A significant CuONP accumulation at the concentration of 0.1 mg/mL was also observed, but the significance was reduced compared to that observed at 0.05 mg/mL. Interestingly, we noticed that late third instar larvae exposed to CuONPs at higher doses, such as 0.1 mg/mL were much smaller than those at 0.05 mg/mL. This suggests that higher doses of CuONPs severely disrupted the metabolism of the larvae, raising the possibility that larvae exposed to the higher doses of CuONPs stopped ingesting food containing CuONPs at early larval stages whereas larvae exposed to lower concentrations continued to ingest food and accumulate CuONPs throughout the entire larval stages. In support of this, we also observed that ingestion of CuONPs causes a dose-dependent decrease in the number of pupa (Figure 2b). Hence, we monitored the egg-to-adult survivorship upon CuONP exposure. The number of F1 flies successfully eclosed to adult was found to be dose-dependently decreased (Figure 2c). In particular, a significant reduction of the survivorship was observed upon higher doses of CuONPs (such as 0.15 mg/mL or 0.25 mg/mL) as compared to untreated control flies. Furthermore, we found that treatment of flies with CuONPs also results in a significant delay in the developmental process of *Drosophila*. As shown in Figure 2d, F1 flies exposed to CuONPs at higher concentrations showed a drastically delayed eclosion compared to those exposed to control or 0.05 mg/mL CuONPs.

These findings are in accord with previous reports that treatment of CuONPs can cause cytotoxicity in *Drosophila*, as well as in other model systems, such as cultured cell lines, C. elegans and marine model species [16,18,21,28]. Notably, contribution of NPs and their released Cu^2+^ leached from the NPs to the overall toxicity of CuONPs is still not clear. For instance, measurement of Cu^2+^ released from CuONPs in cell culture medium suggested that Cu^2+^ cations contribute only to a small extent in CuONP-induced toxicity on HepG2 cells [29]. Another study reported that CuONPs can generate significantly more ROS and DNA damage than dissolved Cu^2+^ ions, suggesting that the surface of NPs plays key roles in inducing toxicity [30]. By contrast, CuONP-associated toxicity was reported to be predominantly mediated by dissolved Cu^2+^ ions in A549 and the lung epithelial BEAS-2B cells [31]. Furthermore, more severe genotoxic effects were detected from dissolved Cu^2+^ ions than those from CuONPs in *Drosophila*, suggesting that Cu^2+^ ions play more important role than physicochemical properties of NPs in inducing toxic effects [15,16]. Hence, more comprehensive analyses with developed techniques are required to differentiate toxicities caused by CuONPs or their dissolved Cu^2+^ ions, which will be of great help to generate hypotheses on how to minimize or abolish the toxic effects of CuONPs and establish a risk assessment of CuONPs via modulating their physicochemical properties, selecting proper route of administration and controlling the release Cu^2+^ ions.

### 3.3. Accumulation of CuONPs in the Gut

**Figure 3:**
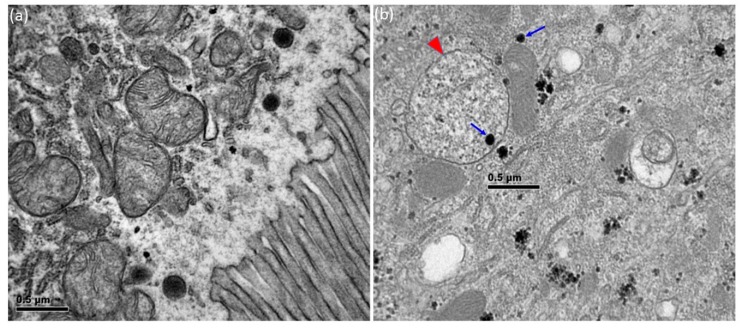
Accumulation of CuONPs in the intestinal epithelial cells. (**a**) CuONPs with the size of 40–50 nm are not detected in the control intestinal epithelial cells; (**b**) CuONPs (arrows) are detected inside the cytoplasm of CuONP-exposed epithelia cells. Note that some of CuONPs are observed inside the vesicle (arrowhead).

NPs are also being used in food industry to improve color, texture and flavor of food [32]. In addition, nanotechnology is now widely accepted and used to improve the delivery of orally administered drugs, implying that the gastrointestinal track is one of the initial organ systems affected by dietary NPs. Indeed, it was suggested that NPs enter and accumulate in the intestine, and subsequently translocate across the intestinal barrier via a few possible mechanisms, including endocytosis, persorption, and putative para-cellular uptake of NPs [33]. Furthermore, a previous study using *Drosophila* suggested that CuONPs can travel inside the midgut epithelial cells and cross over the intestinal barrier to interact with circulating hemolymph, which is equivalent to the blood in mammals [16]. We thus examined the distribution and localization of NPs in the midgut of third instar larvae fed with CuONPs by TEM after ultrathin sectioning. In the control intestinal epithelial cells, CuONPs with the size of 40–50 m were not detected (Figure 3a). However, in the CuONP-exposed epithelial cells CuONPs were found in the cytosol, as well as inside the vesicle (Figure 3b).

### 3.4. CuONPs Induce Cell Death and Oxidative Stress in the Gut

**Figure 4:**
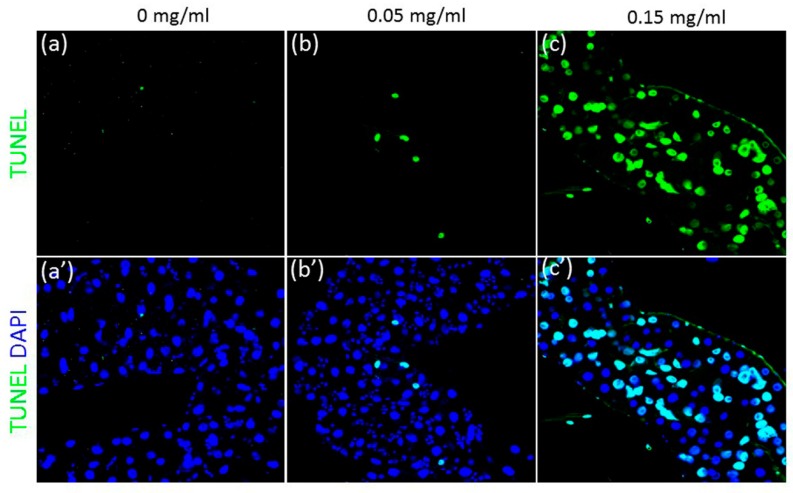
CuONP exposure causes apoptosis of the gut epithelia cells. Flies were exposed to CuONPs at the concentration of (**a**,**a’**) 0 mg/mL, (**b**,**b’**) 0.05 mg/mL and (**c**,**c’**) 0.15 mg/mL. The third instar larval guts show a dose-dependent increase in the number of terminal deoxynucleotidyl transferase dUTP nick end labelling (TUNEL)-positive epithelial cells (green).

Toxicological effects of particles, such as AgNPs and ZnONPs in the human intestinal cell line Caco-2 were reported. NP exposure was found to induce mitochondrial and DNA damage, cell membrane leakage and inflammation, resulting in cell-cycle arrest and subsequently cell death [34,35,36]. Since we observed a significant decrease in viability and delay in development upon CuONP exposure (Figure 2c,d), we examined whether exposure to CuONPs can cause cell death of the intestinal epithelial cells by performing TUNEL assay. In the untreated gut, TUNEL-positive epithelial cells were barely detected (Figure 4a,a’). However, exposure to CuONPs resulted in a dose-dependent increase in the number of TUNEL-positive cells in the midgut, suggesting that the gut epithelial cells underwent apoptosis upon CuONPs (Figure 4b,b’,c,c’). Interestingly, it was shown that NPs, such as AgNPs, TiO_2_NPs and SiO_2_NPs are known to disrupt the gut microbiota in the mouse model [37,38,39]. Furthermore, dietary exposure to AgNPs resulted in an increase in Gram-positive genera, in particular Lactobacillus, in the midgut of *Drosophila*, and toxicity of NPs within the gut was considered to be responsible for developmental delay and decreased survivorship [40], suggesting that CuONPs may not only induce apoptosis of epithelial cells, but also disrupt microbiota in the gut, leading to poor survivorship and delayed development.

**Figure 5:**
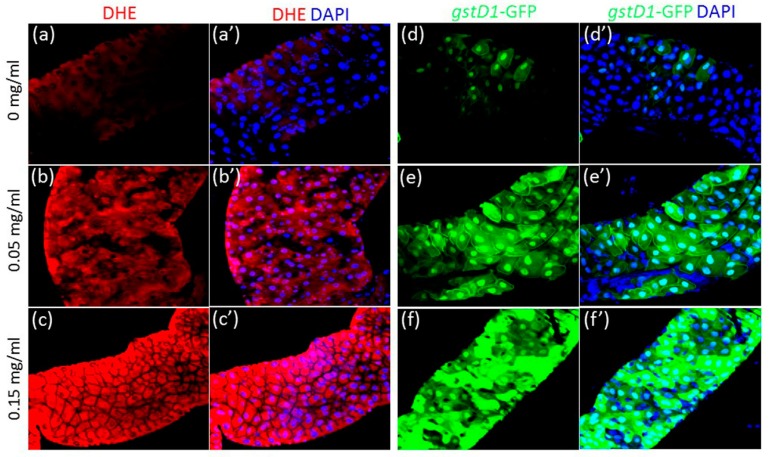
CuONPs induce excessive reactive oxygen species (ROS) production in the gut. Flies were exposed to CuONPs at the concentration of (**a**,**a’**,**d**,**d’**) 0 mg/mL, (**b**,**b’**,**e**,**e’**) 0.05 mg/mL and (**c**,**c’**,**f**,**f’**) 0.15 mg/mL. (**a**,**a’**) Dihydroethidium (DHE) staining that detects superoxide levels shows the basal levels of ROS in the control gut epithelial cells. (**b**,**b’**,**c**,**c’**) ROS levels become gradually increased upon CuONPs in a dose-dependent manner. Transgenic flies carrying the oxidative stress reporter gene GstD1-GFP were used to monitor the effects of CuONPs on intracellular ROS induction. (**d**,**d**’) The control gut shows weak GFP expression. (**e**,**e’**,**f**,**f’**) GFP expression is dose-dependently increased upon CuONPs.

Oxidative stress is considered to be one of the primary causes of cytotoxicity, genotoxicity, inflammation, cell death and altered nutrient absorption induced by NP exposure in the gut [41,42,43]. We have also previously shown that AgNP ingestion causes excessive intracellular ROS induction in the *Drosophila* testis, leading to oxidative stress responsible for defects in germline stem cell homeostasis and a significant decrease in male fecundity [44]. All these observations suggest that NP-induced oxidative stress may cause a defect in the function of macromolecules, such as protein and lipid, leading to its detrimental toxic effects on various organ systems. Hence, we examined the effects of CuONPs on ROS induction in the gut of third instar larvae. We found that CuONPs greatly increase ROS levels. Dihydroethidium (DHE) probe was used to monitor ROS, particularly superoxide (O2-) levels, as it readily reacts with superoxide anions to form 2-hydroxyethidium, generating red fluorescence [45]. In the control gut of larvae exposure to control CuONPs, basal levels of ROS were detected in the gut epithelial cells (Figure 5a,a’). However, a dose-dependent increase in DHE expression was detected upon CuONP exposure (Figure 5b,b’,c,c’). To further confirm the effects of CuONPs on ROS induction, we next used transgenic flies carrying the independent oxidative stress reporter gene GstD1-GFP and assessed whether CuONP exposure can enhance the reporter activity in the gut epithelial cells. Upon oxidative stress, the transcriptional factor Nrf2, possibly together with small-Maf, binds to antioxidant response element (ARE) within the promoter region of the antioxidant gene GstD1, leading to an increase in the expression of GstD1 [46]. Hence, high levels of GFP expression are expected upon oxidative stress. In accordance with the findings obtained from DHE staining, only basal levels of GFP were detected in the control gut epithelial cells (Figure 5d,d’). However, we observed a dose-dependent increase in GFP expression in the epithelial cells upon CuONP exposure (Figure 5e,e’,f,f’). These observations strongly suggest that ingested CuONP induced oxidative stress, which is closely associated with apoptosis of the gut epithelial cells, and decreased viability and delayed development of the organism.

### 3.5. Inhibition of Nrf2 Further Decreases the Poor Survivorship Caused by CuONPs

**Figure 6:**
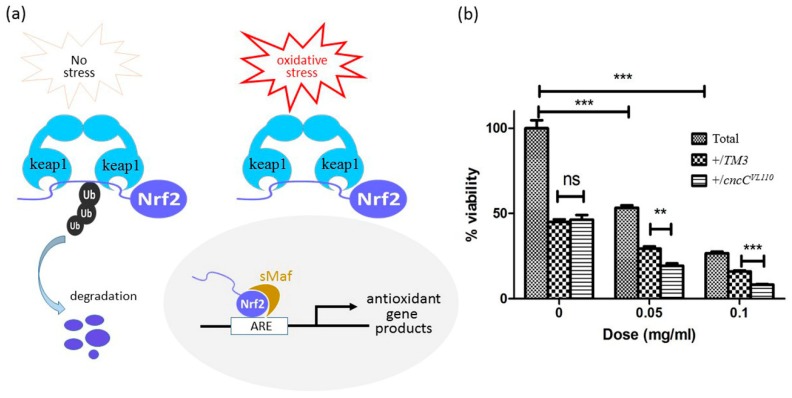
ROS levels modulate the toxic effects caused by CuONPs. (**a**) Schematic diagram suggesting the role of Nrf2 in the regulation of antioxidant gene expression; (**b**) Introducing one mutant allele of CncC (the *Drosophila* homolog of Nrf2) further decreased the poor survivorship caused by CuONP exposure. Error bar = SEM, ** *p*-value < 0.01; *** *p*-value < 0.001.

To demonstrate that the decreased viability in CuONP-treated flies was at least in partly associated with excessive ROS induction, we examined the effects of loss-of-function of Nrf2 on the decreased viability. Keap1/Nrf2 complex plays as an important cellular sensor for oxidative stress [47,48]. Under the normal condition, the transcriptional factor Nrf2 is involved in the cellular response to oxidative and electrophilic stress [49], and is negatively regulated by Keap1 via ubiquitination in the cytoplasm [50]. However, upon oxidative stress by oxidants or electrophilic insults, Nrf2 becomes activated by protein stabilization, translocates into the nucleus to engage in the transcriptional activation of genes encoding a large pool of antioxidant and phase II detoxifying enzymes, including superoxide dismutase (SOD), catalase and glutathione peroxidase (GTPx), to scavenge excessive intracellular ROS (Figure 6a). Thus, we reasoned that CuONP-induced fly lethality would be even more severe when the activity of Nrf2 is down-regulated. Nrf2 activity was genetically manipulated in both control and CuONP-fed F1 progenies by introducing one copy of CncC (the *Drosophila* homolog of Nrf2) mutant alleles (CncCK6), and the decreased viability caused by CuONPs was monitored. As expected, CuONP-induced poor viability was further decreased in CncC+/− heterozygous flies compared to control flies (Figure 6b). This provides compelling evidence that oxidative stress is one of the direct causes of CuONP-induced toxicity and that antioxidants or detoxifying enzymes play essential roles in salvaging ROS-associated toxicity upon CuONP exposure in *Drosophila*.

## 4. Conclusions

The oral route of CuONP administration has resulted in a significant toxicity in *Drosophila* at the organism level. Specifically, CuONP exposure caused a decline in egg-to-adult survivorship and a delay in development of CuONP-fed offsprings. Furthermore, CuONPs induced a drastic ROS production in the *Drosophila* gut, which was possibly related to an increase in apoptosis of the gut epithelial cells. Importantly, more significant decreased viability was observed in CncC+/− flies compared to that of in control flies (carrying a balancer allele) upon CuONPs, indicating the essential role of ROS levels in CuONP-mediated toxicity in vivo. Nonetheless, limited understanding of the mechanisms of CuONP-associated toxicity would need further elucidation to have meaningful assessment of nanosafety. Furthermore, comprehensive understanding of underlying mechanisms is of great importance to assess the environmental risk of CuONPs and to expand their use safely.
